# Interaction mode of CIDE family proteins in fly: DREP1 and DREP3 acidic surfaces interact with DREP2 and DREP4 basic surfaces

**DOI:** 10.1371/journal.pone.0189819

**Published:** 2017-12-14

**Authors:** Chang Min Kim, Sun Hee Jeon, Jun-Hyuk Choi, Jun Hyuck Lee, Hyun Ho Park

**Affiliations:** 1 School of Natural Science, Department of Chemistry and Biochemistry and Graduate School of Biochemistry, Yeungnam University, Gyeongsan, Republic of Korea; 2 Department of Metrology for Quality of Life, Center for Bioanalysis, Korea Research Institute of Standards and Science, Daejeon, Republic of Korea; 3 Unit of Polar Genomics, Korea Polar Research Institute, Inchon, Republic of Korea; Weizmann Institute of Science, ISRAEL

## Abstract

Cell death-inducing DNA fragmentation factor 45 (DFF45)-like effector (CIDE) domains were initially identified as protein interaction modules in apoptotic nucleases and are now known to form a highly conserved family with diverse functions that range from cell death to lipid homeostasis. In the fly, four CIDE domain-containing proteins (DFF-related protein [DREP]-1–4) and their functions, including interaction relationships, have been identified. In this study, we introduced and investigated acidic side-disrupted mutants of DREP1, DREP2, and DREP3. We discovered that the acidic surface patches of DREP1 and DREP3 are critical for the homo-dimerization. In addition, we found that the acidic surface sides of DREP1 and DREP3 interact with the basic surface sides of DREP2 and DREP4. Our current study provides clear evidence demonstrating the mechanism of the interactions between four DREP proteins in the fly.

## Introduction

**Fig 1:**
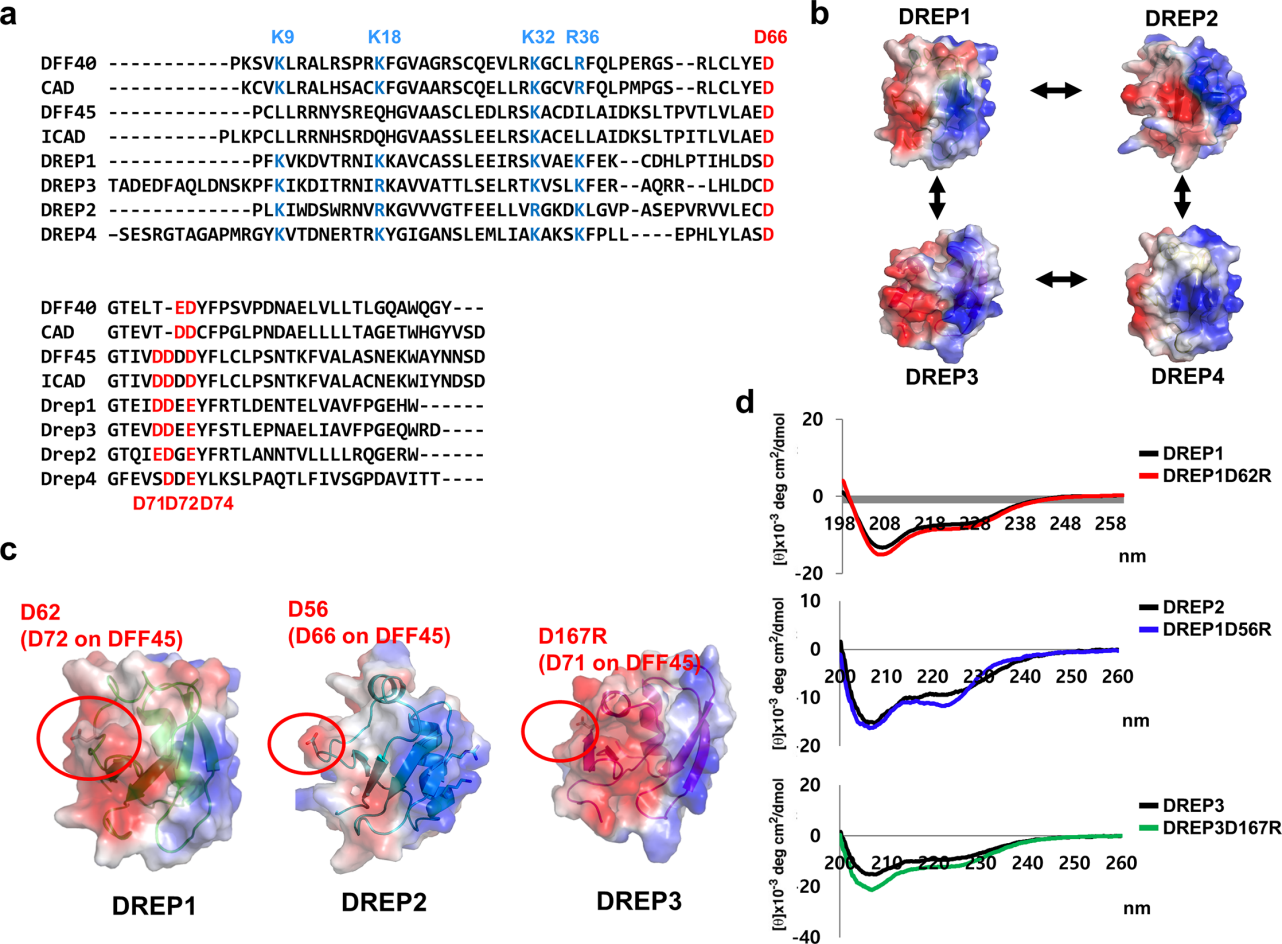
Surface feature of cell death-inducing DNA fragmentation factor 45 (DFF45)-like effector (CIDE) domain and generation of mutation on acidic surface of CIDE domains of DFF-related protein (DREP) family of proteins (a) Sequence alignment of CIDE domains. Residues conserved and involved in interaction between DFF40 and DFF45 are indicated in blue and red for basic and acidic residues, respectively, and conserved residues are similarly indicated. (b) Electrostatic surfaces generated using modeled structures are shown. Arrows indicate previously identified interactions between DREP family proteins. Electrostatic surfaces were calculated using PyMOL. (c). Location of mutation position on CIDE domains of DREP1, DREP2, and DREP3. Red circles indicate mutation residues. (d). Circular dichroic (CD) spectra of wildtype and mutant CIDE domains.

The cell death-inducing DNA fragmentation factor 45 (DFF45)-like effector (CIDE) family of proteins contains CIDE domains for protein interaction and in mammals, consists of five proteins, including DFF40 (caspase-activated DNase [CAD] in mouse), DFF45 (inhibitor of CAD [ICAD] in mouse), CIDE-A, CIDE-B, and CIDE-C (fat-specific protein 27 [FSP27] in mouse) [1]. The general feature of the CIDE domain is a conserved sequence containing conserved basic residues at the N-terminus and acid residues at the C-terminus (Fig 1A). The conserved charged patches are critical for homo- and hetero-oligomerization. Although all CIDE family proteins were initially identified as regulators of apoptosis, other functions of CIDE family proteins in lipid droplet formation and energy metabolism have recently been suggested by genetic studies [2,3]. The representative CIDE family proteins are DNA fragmentation factors (DFFs), DFF40 and DFF45, which control the apoptotic DNA fragmentation that is the biochemical hallmark of apoptotic cells [4,5]. DFF40 is a nuclease whereas DFF45 is an inhibitor that suppresses the nuclease activity of DFF40 by a tight interaction with the CIDE domain [5,6]. During apoptosis, DFF40 is activated by dissociation from DFF45, which is cleaved by activated effector caspases [7,8]. CIDE domain-mediated DFF40 oligomerization is important for the activity of the enzyme [9]. CIDE-A, CIDE-B, and CIDE-C proteins, which have conserved CIDE domains initially identified as apoptosis-related proteins because the ectopic expression of CIDE-A, CIDE-B, and CIDE-C induced apoptosis and DNA fragmentation in various human cell lines [10,11]. However, interestingly, recent genetically modified mouse studies showed that CIDE family proteins were more likely linked to the energy metabolism and size control of lipid droplets [2,3]. For example, overexpression of FSP27 induced the accumulation of lipid droplets and increased the droplet size of adipocytes whereas its knockdown produced opposite results [3]. Several knock-in and knock-out mouse studies also supported the critical role of CIDE family proteins, including CIDE-A, CIDE-B, and CIDE-C proteins, in lipid synthesis and energy metabolism [12–14].

In *Drosophila melanogaster*, four conserved CIDE domain-containing proteins, DFF-related protein 1 (DREP1)–DREP4, have been identified [15]. DREP1 and DREP4 have been identified as DFF45 and DFF40 homologs, respectively, whereas the functions of DREP2 and DREP3 are vague [16,17]. A recent *in vitro* biochemical study suggested that DREP2 might be another apoptotic nuclease that is inhibited by DREP3 by interacting with the CIDE domain [18]. Most recently, helical assembly of CIDE domains of DREP2 and DREP4 [9], and a cyclic interaction between DREP proteins, which shows that DREP1 interacts with DREP4 and DREP2, DREP2 interacts with DREP1 and DREP3, and DREP3 interacts with DREP2 and DREP4, have been proposed, but the biological implications of the cyclic interaction have not yet been elucidated (Fig 1B) [19,20]. All DREP interactions are mediated by the CIDE domain, which is a dynamic protein interaction module. Structural and mutagenesis studies of the CIDE domain including hetero- and homodimeric complex structures showed that the surface feature of the CIDE domain could be divided into two distinctly charged sides, which are the basic and acidic surface sides (Fig 1B) [19,21–26]. The CIDE domain can form a homo- or hetero-dimer, using the basic surface side of one CIDE domain and the acidic surface side of another, which might be the general mode of CIDE:CIDE interactions [26]. Our previous mutagenesis study showed that DREP2 CIDE uses the basic surface side to interact with DREP1 CIDE and DREP3 CIDE [19]. To further elucidate the mode of the CIDE domain-mediated interactions of all DREP proteins in the fly system, we performed biochemical and mutational studies using acidic side-disrupted mutants of DREP1 (DREP1D62R), DREP2 (DREP2D56R), and DREP3 (DREP3D167R) (Fig 1C). In this study, we found that acidic surface patches of DREP1 and DREP3 were critical for the homo-dimerization. We also found that DREP1 and DREP3 used the acidic surface side to interact with the basic surface side of DREP2 and DREP4. Our current study provides clear evidence demonstrating the mode of interaction between four DREP proteins in fly.

## Materials and methods

### Sequence alignment

The amino acid sequence of CIDEs was analyzed using the Clustal Omega program (http://www.ebi.ac.uk/Tools/msa/clustalo/).

### Homology modeling of CIDE domain structures

The methods used for homology modeling of DREP1 CIDE, DREP2 CIDE, and DREP4 CIDE were previously reported elsewhere [19,20]. Briefly, the CIDE-A structure (PDBid: 2EEL) was used as a template for DREP2 CIDE. The CIDE B structure (PDBid: 1D4B) was used for DREP1 CIDE and DREP4 CIDE. Electrostatic surfaces and ribbon diagrams were generated using the PyMOL program (*The PyMOL Molecular Graphics System*, DeLano Scientific, San Carlos).

### Mutagenesis

Site-directed mutagenesis was conducted using a Quick-change mutagenesis kit (Stratagene) according to the manufacturer’s protocols. Mutagenesis was then confirmed by sequencing.

### Target protein expression and purification

The DREP2, DREP3, and DREP4 CIDE domains (amino acid residues: 1–84, 111–195, and 39–130, respectively) were expressed and purified as described in previous studies [18,27]. The DREP1 CIDE (amino acid residues: 10–90) was newly cloned into a pOKD home-made vector modified from pET24d vector (Fisher Scientific). All four CIDE proteins were purified using the same method described in our previous studies [27,28]. The plasmids containing the CIDE domains of all four proteins were transformed into BL21 (DE3) *Escherichia coli* competent cells (Sigma-Aldrich), and then expression was induced by treating the bacteria with 0.5 mM isopropyl β-D-thiogalactopyranoside (IPTG) overnight at 20°C. The bacteria were then collected, resuspended, and lysed by sonication in 50 mL lysis buffer (20 mM Tris-hydrochloride [HCl] pH 7.9, 500 mM sodium chloride [NaCl], and 10 mM imidazole). Next, the bacterial lysate was centrifuged, the supernatant was subsequently collected, and then applied to a gravity-flow column (Bio-Rad) packed with Ni-NTA affinity resin (Qiagen). The unbound bacterial proteins were removed from the column by washing with buffer (20 mM Tris-HCl pH 7.9, 500 mM NaCl, 60 mM imidazole, and 10% glycerol), and then the target proteins were eluted using an elution buffer (20 mM Tris-HCl pH 7.9, 500 mM NaCl, and 250 mM imidazole). The protein was further purified using a Superdex 200 gel-filtration column (GE Healthcare). All the mutant proteins were expressed and purified by a method similar to that used to purify the wildtype CIDE domains.

### Circular dichroism (CD) spectroscopy

The secondary structures were measured by circular dichroism (CD) spectroscopy, using a J-715 spectropolarimeter at Korea Basic Science Institute in South Korea. The spectra were obtained from 200 to 260 nm at 25°C in a 0.1-cm path-length quartz cuvette at a bandwidth of 1.0 nm, speed of 50 mm/min, and 5-s response time. The protein samples in buffer containing 20 mM Tris-HCl at pH 8.0 and 150 mM NaCl were diluted to 0.1 mg/mL prior to use. Three scans were accumulated, averaged, and used for the data analysis.

### Multi-angle light scattering (MALS)

The molar mass of DREP1 D62R and DREP3D167R mutants were determined by multi-angle light scattering (MALS). Target proteins were injected into a Superdex 200 HR 10/30 gel filtration column (GE Healthcare) that had been equilibrated in a buffer containing 20 mM Tris at pH 8.0 and 150 mM NaCl. The chromatography system was coupled to a three-angle light scattering detector (mini-DAWN EOS) and a refractive index detector (Optilab DSP) (Wyatt Technology). Data were collected every 0.5 s at a flow rate of 0.2 mL/min and analyzed using the ASTRA program, which gave the molar mass and mass distribution (polydispersity) of the sample.

### Complex assay by size-exclusion chromatography

For size-exclusion chromatography analysis to detect complex formation, the purified target proteins were mixed and placed in a size-exclusion column (Superdex 200 HR 10/30, GE Healthcare) that was pre-equilibrated with 20 mM Tris-HCl (pH) 8.0 and 150 mM NaCl. The peak fractions were collected and subjected to sodium dodecyl sulfate-polyacrylamide gel electrophoresis (SDS-PAGE).

### Native PAGE shift assay

The protein interactions between the CIDE domains and various mutants were analyzed by native (non-denaturing) PAGE using a PhastSystem (GE Healthcare) with pre-made 8–25% acrylamide gradient gels (GE Healthcare). The separated and purified proteins were pre-incubated at room temperature for 1 h before loading onto the gel, and Coomassie Brilliant Blue was used to stain and detect the shifted bands.

## Results and discussion

### Surface feature of CIDE domain and mutation of acidic surface side of DREP1, DREP2, and DREP3 CIDE domains based on sequence alignment

DFF40 and DFF45 interaction via the CIDE domain and FSP27 CIDE domain-mediated homo-dimerization are critical to apoptotic DNA fragmentation and lipid droplet formation, respectively [23,25,26]. Structural analysis of the heterodimeric structure of CIDE domains and the interaction between DFF40 and DFF45 and the homo-dimeric structure of the FSP27 CIDE domain shows that the basic surface patch of one CIDE domain interacts with the acidic surface patch of another CIDE domain [23,25,26]. Sequence alignment showed that the overall features of each electrostatic surface of the DREPs were very similar to those of DFF family, in that CIDE domain is divided by two distinct sides, acidic and basic side [19,20] (Fig 1A and 1B). K9, K18, K32, and R36 on DFF40 CIDE and D66, D71, D72 and D74 on DFF45 CIDE, which are the important residues involved in their interactions, are conserved in the CIDE domains of DREP1, DREP2, DREP3, and DREP4 (Fig 1A). Although a previous study showed the cyclic interaction between the DREP family: DREP1-DREP2-DREP3-DREP4-DREP1 [20], the mechanism by which certain DREPs accommodate two DREP family members has not been identified and which side of DREP CIDE, acidic side or basic side, are used to interact with their binding partner (Fig 1B).

As a first step in elucidating the interaction mode between the DREP family members, we generated acidic side-disrupted mutants of DREP1 (DREP1D62R), DREP2 (DREP2D56R), and DREP3 (DREP3D167R) based on the sequence alignment (Fig 1C). D62 on the acidic surface side of DREP1 CIDE, which corresponded to D72 on DFF45 and might be critical to the interaction, was mutated to include an oppositely charged arginine (DREP1D62R). For DREP2 and DREP3, D56 (which corresponded to D66 on DFF45) on DREP2 CIDE and D167R (which corresponded to D71 on DFF45) on DREP3 CIDE were mutated to arginine. Before analyzing the interactions in vitro, we confirmed that the mutant structures were not altered by mutagenesis, using their far ultraviolet (UV) CD spectra (Fig 1D). As shown in Fig 1D, the mutants and wildtype showed similar CD spectral patterns typical of α-helix and β-sheet mixed proteins with two pronounced minima at 208 and 222 nm, indicating that the mutant structures were not changed by mutagenesis and were still functional (Fig 1D).

### Mutation of acidic surface of CIDE domains of DREP1 and DREP3 disrupts homodimerization

**Fig 2:**
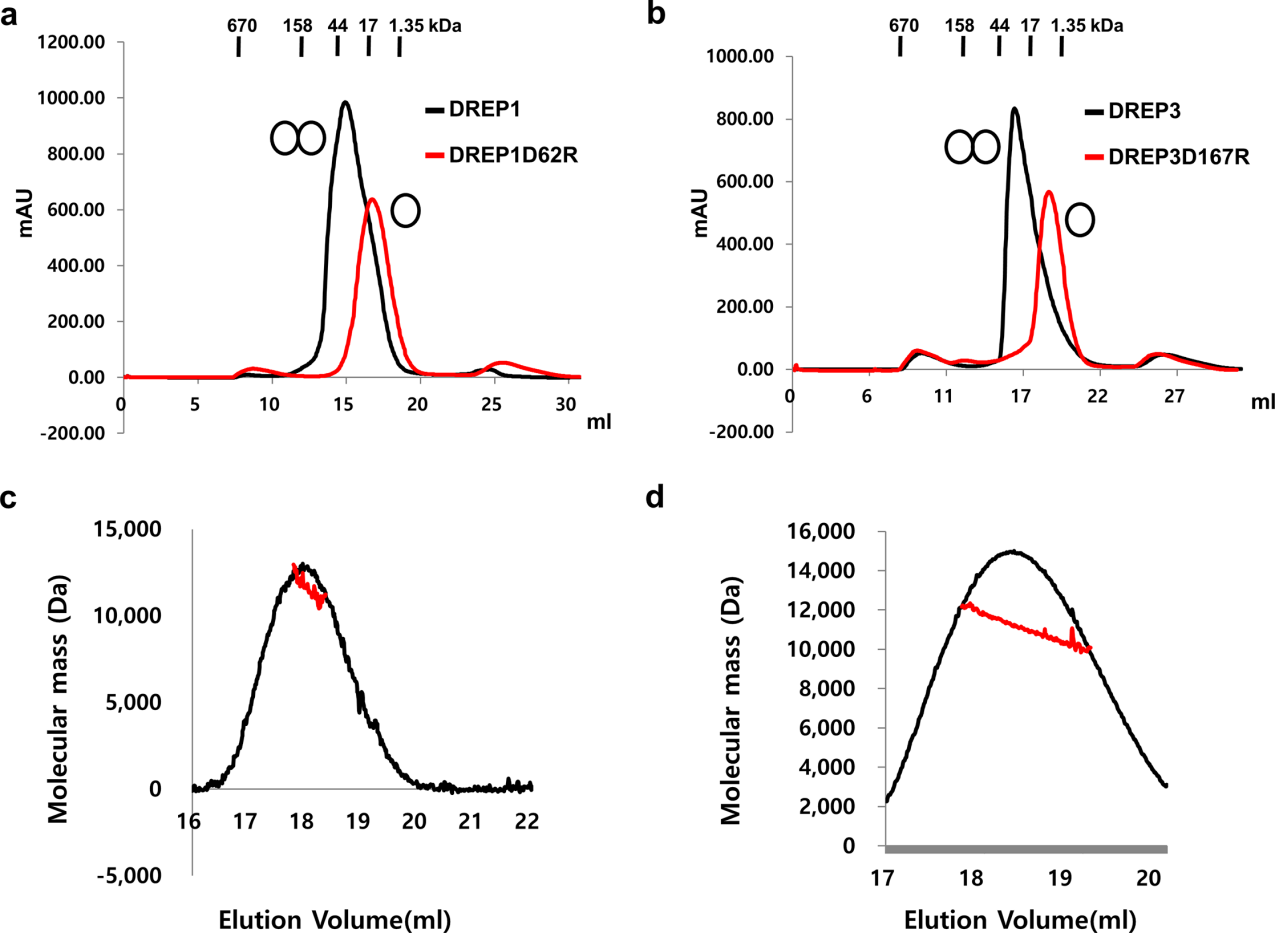
Mutation on acidic surface side of cell death-inducing DNA fragmentation factor 45 (DFF45)-like effector (CIDE) domains of DFF-related protein 1 (DREP1) and DREP3 disrupt homo-dimerization (a). Size-exclusion chromatograms of wild-type (DREP1) and mutant (DREP1D62R). Black and double black circles indicate monomer and dimer, respectively. (b). Size-exclusion chromatograms of DREP3 and its mutant, DREP3D167R. Determination of molar masses of (c) DREP1D62R and (d) DREP3D167R using multi-angle light scattering (MALS).

The CIDE domain is a highly dynamic protein interaction module that exists in various states in solution [19,20]. DREP1 CIDE and DREP3 CIDE have been reported to exist as dimers in solution while DREP2 CIDE and DREP4 CIDE formed highly oligomeric state [20]. To analyze the effect of the mutation on homodimerization and homo-oligomerization, we initially performed size-exclusion chromatography to compare the molecular size of each wild-type with that of its mutant, which was disrupted on the acidic surface. As shown in Fig 2A and 2B, in both cases, the molecular size decreased following the introduction of the mutation, indicating that the mutation of the acidic surface of the CIDE domain of DREP1 and DREP3 failed to form a homodimer. We further analyzed the molecular weight of each DREP1 and DREP3 CIDE domain mutant to understand the effects of the stoichiometry change, using a MALS experiment. The theoretical monomeric molecular weights of DREP1D62R and DREP3D167R, including the C-terminal His-tag, were 10,565 and 10,804 Da respectively. The experimentally calculated molecular weights in the MALS were 10.825 and 11,150 Da, with a 0.4 and 2.4% fitting error for DREP1D62R and DREP3D167R, respectively. This observation indicates that the acidic surface of the CIDE domains of DREP1 and DREP3 are crucial to their homo-dimerization.

### Acidic surface of DREP1 CIDE is involved in interaction with DREP4 CIDE and DREP2 CIDE

**Fig 3:**
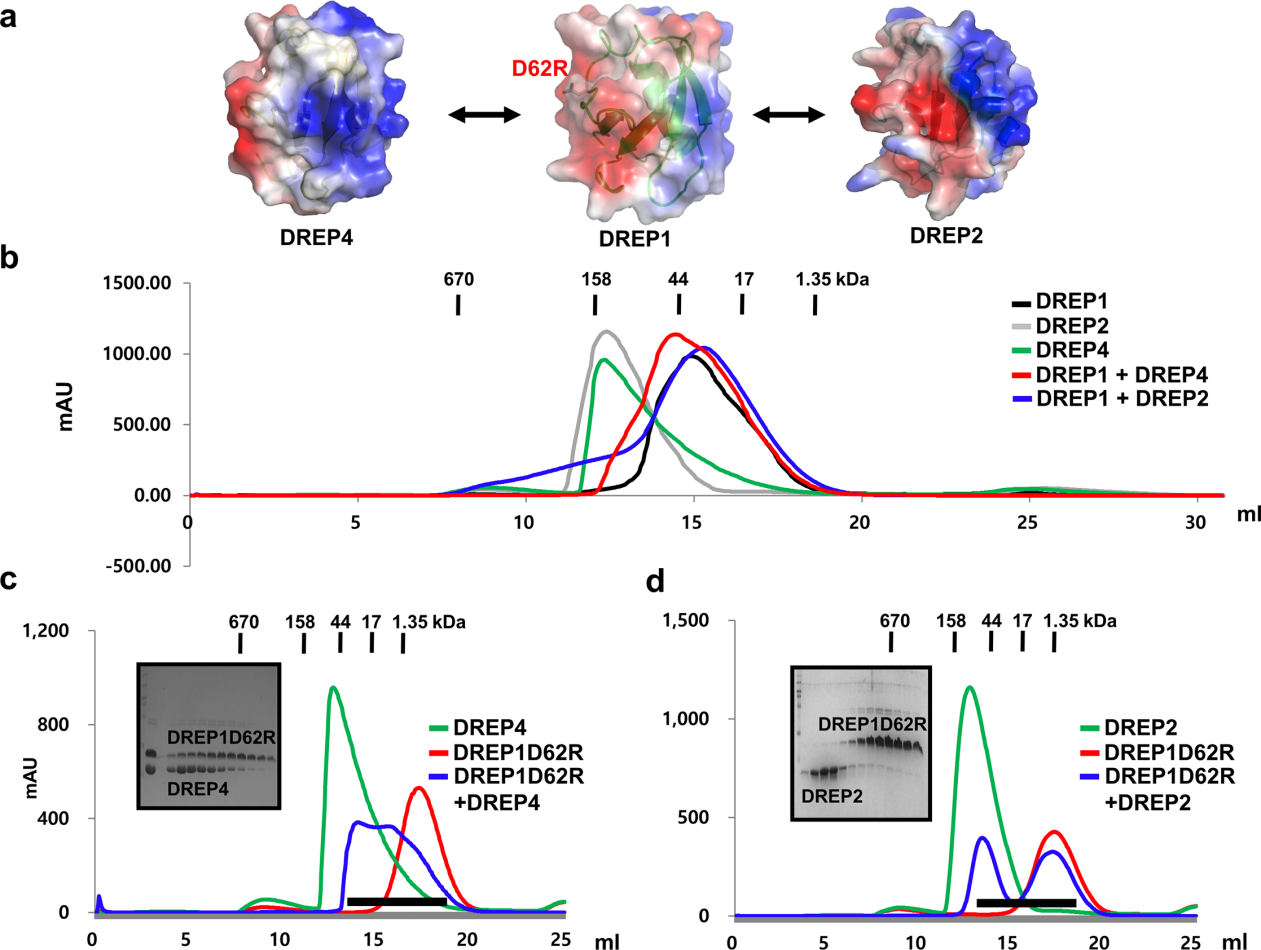
Acidic surface-disrupted mutant of DNA fragmentation factor 45 (DFF45)-related protein 1 (DREP1) cell death-inducing DFF45-like effector (CIDE), DREP1D62R, did not bind to DREP4 CIDE and DREP2 (a) Known interaction partners of DREP1. (b) Size-exclusion chromatograms of CIDE domains of DREP1, DREP2, and DREP4, and mixtures of their CIDE domains. (c) Size-exclusion chromatograms of DREP4 CIDE, DREP1D62R, and their mixture. Sodium dodecyl sulfate-polyacrylamide gel electrophoresis (SDS-PAGE)-loaded fractions (black bar) of DREP1D62R and DREP4 CIDE mixture from size-exclusion chromatography are shown on left side. (d) Size-exclusion chromatograms of DREP2 CIDE, DREP1D62R, and their mixture. SDS-PAGE-loaded fractions (black bar) of DREP1D62R and DREP2 CIDE from size-exclusion chromatography are shown on left side.

We analyzed the interaction between the DREP1D62R mutant and the two previously identified binding partners, DREP4 and DREP2 (Fig 3A). Before the analysis, we confirmed the interaction of the wildtype DREP1 CIDE with the CIDE domains of DREP2 and DREP4. In the size-exclusion chromatographic analysis, DREP4 CIDE and DREP2 CIDE formed a highly oligomeric homo-complex in a solution that eluted at approximately 12–14 mL while DREP1 CIDE formed a dimer in a solution that eluted at approximately 15–17 mL (Fig 3B). In the size-exclusion analysis, mixing the wildtype DREP1 CIDE with the DREP2 CIDE or DREP3 CIDE formed a heterodimer in a solution that eluted at approximately 15–17 mL, indicating that the wildtype DREP1 CIDE tightly interacted with both DREP4 CIDE and DREP2 CIDE (Fig 3C). Then, we analyzed the interaction of DREP1D62R, which was disrupted on its acidic surface, with DREP4 CIDE and DREP2 CIDE by size-exclusion chromatography, followed by SDS-PAGE. As shown in Fig 3B, the oligomeric DREP4 CIDE and DREP2 CIDE peaks disappeared after they formed a heterodimer with DREP1 CIDE and a complex peak was observed around 15–17 mL in the size-exclusion column. However, after DREP4 CIDE and the DREP1D62R mutant were combined, the oligomeric peak of DREP4 CIDE did not disappear, indicating that DREP4 CIDE did not form a stable complex with DREP1D62R (Fig 3C). The SDS-PAGE showed two proteins that migrated separately, supporting the data from the size-exclusion chromatography (Fig 3C). After analyzing the interaction between DREP1D62R and DREP4 CIDE, we investigated whether the mutant interacted with DREP2 CIDE. To accomplish this, we performed size-exclusion chromatography again, followed by SDS-PAGE, and checked the position of the peaks and co-migration. A complex also dissociated as shown by the size-exclusion chromatography and SDS-PAGE (Fig 3D), indicating that the acidic surface of DREP1 CIDE interacted with both DREP2 and DREP4.

### Acidic surface side of DREP2 CIDE is not critical for the interaction with DREP1 CIDE and DREP3 CIDE

**Fig 4:**
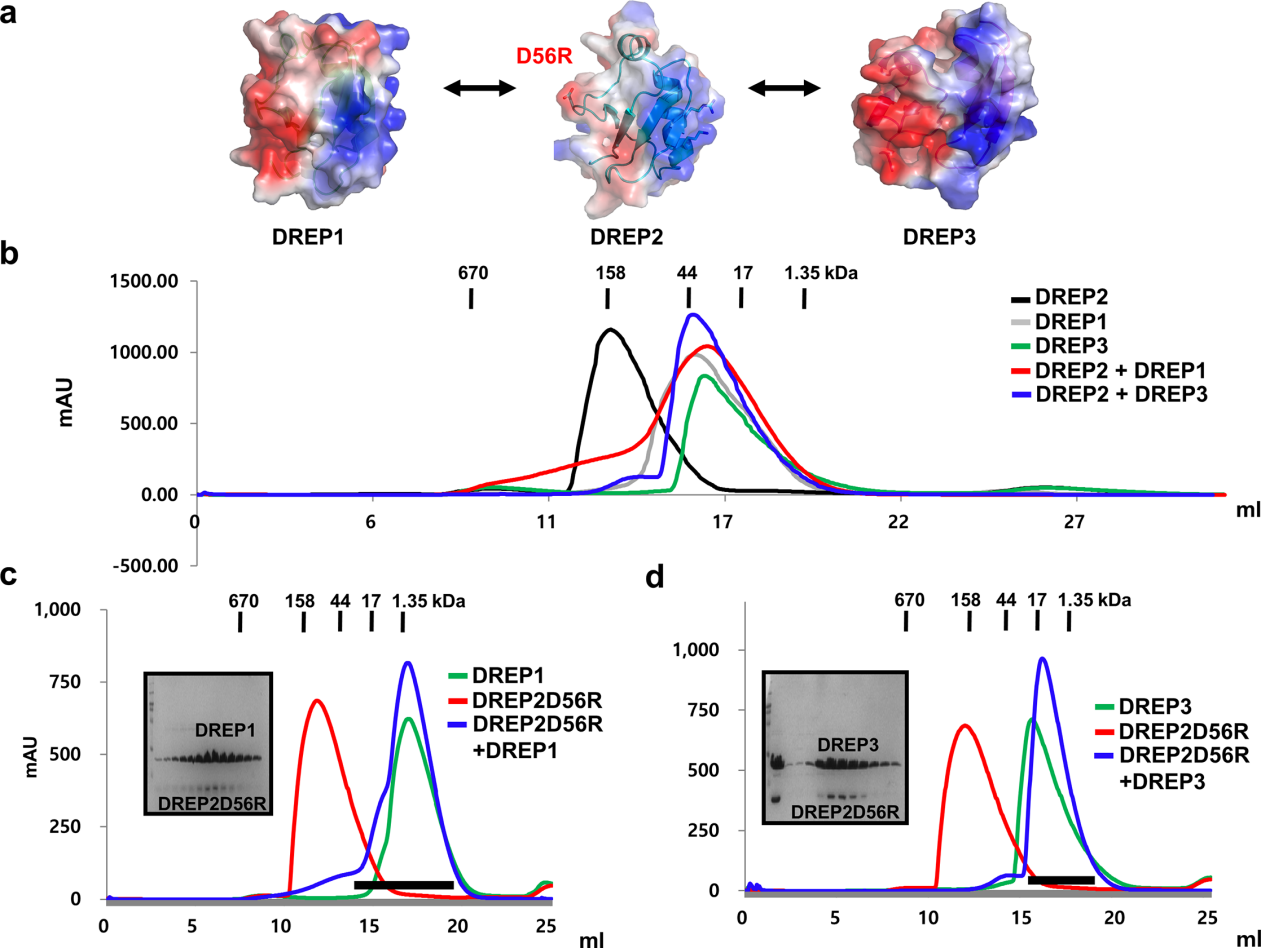
Acidic surface side of DNA fragmentation factor 45 (DFF45)-related protein 2 (DREP2) cell death-inducing DFF45-like effector (CIDE) is not critical for interaction with DREP1 CIDE and DREP3 CIDE (a) Known interaction partners of DREP2. (b) Size-exclusion chromatograms of CIDE domains of DREP1, DREP2, and DREP3, and mixtures of their CIDE domains. (c) Size-exclusion chromatograms of DREP1 CIDE, DREP2D56R, and their mixture. Sodium dodecyl sulfate-polyacrylamide gel electrophoresis (SDS-PAGE)-loaded fractions (black bar) of mixture of DREP2D56R and DREP1 CIDE from size-exclusion chromatography are shown on left side. (d) Size-exclusion chromatograms of DREP3 CIDE, DREP2D56R, and their mixture. SDS-PAGE-loaded fractions (black bar) of mixture of DREP2D56R and DREP2 CIDE from size-exclusion chromatography are shown on left side.

The interaction mode of DREP2 CIDE with DREP1 CIDE and DREP3 CIDE was also investigated by a method similar to that used to examine the DREP1 CIDE interactions (Fig 4A). Previously identified interactions of DREP2 CIDE with DREP 1 CIDE and DREP3 CIDE were also confirmed by size-exclusion chromatography. The binding of the oligomeric DREP2 CIDE to DREP1 CIDE or DREP3 CIDE formed a heterodimer in a solution that eluted around 16–17 mL in the size-exclusion chromatography (Fig 4B). This indicates that the wildtype DREP2 CIDE tightly interacted with both DREP1 CIDE and DREP3 CIDE. After analyzing the position of the eluted peaks in the size-exclusion chromatography profiles, we analyzed the mutation effects. DREP2D56R is disrupted on the acidic surface of DREP2 CIDE, and the profiles of the size-exclusion chromatography and SDS-PAGE results showed that it produced complex bands when it was mixed with DREP1 CIDE or DREP3 CIDE, indicating that DREP2D56R still binds to DREP1 CIDE and DREP3 CIDE (Fig 4C and 4D). This result supports the notion that the acidic surface of DREP1 interacts with the basic surface of DREP2, and the acidic surface of DREP2 CIDE is not involved in the interaction with DREP1 CIDE. This result also indicates that the acidic surface of DREP2 CIDE is not critical for the interaction with DREP3 CIDE. Therefore, the basic surface of DREP2 interacted with the acidic surfaces of both DREP1 CIDE and DREP3 CIDE.

### Acidic surface of DREP3 CIDE is involved in interaction with DREP2 CIDE and DREP4 CIDE

**Fig 5:**
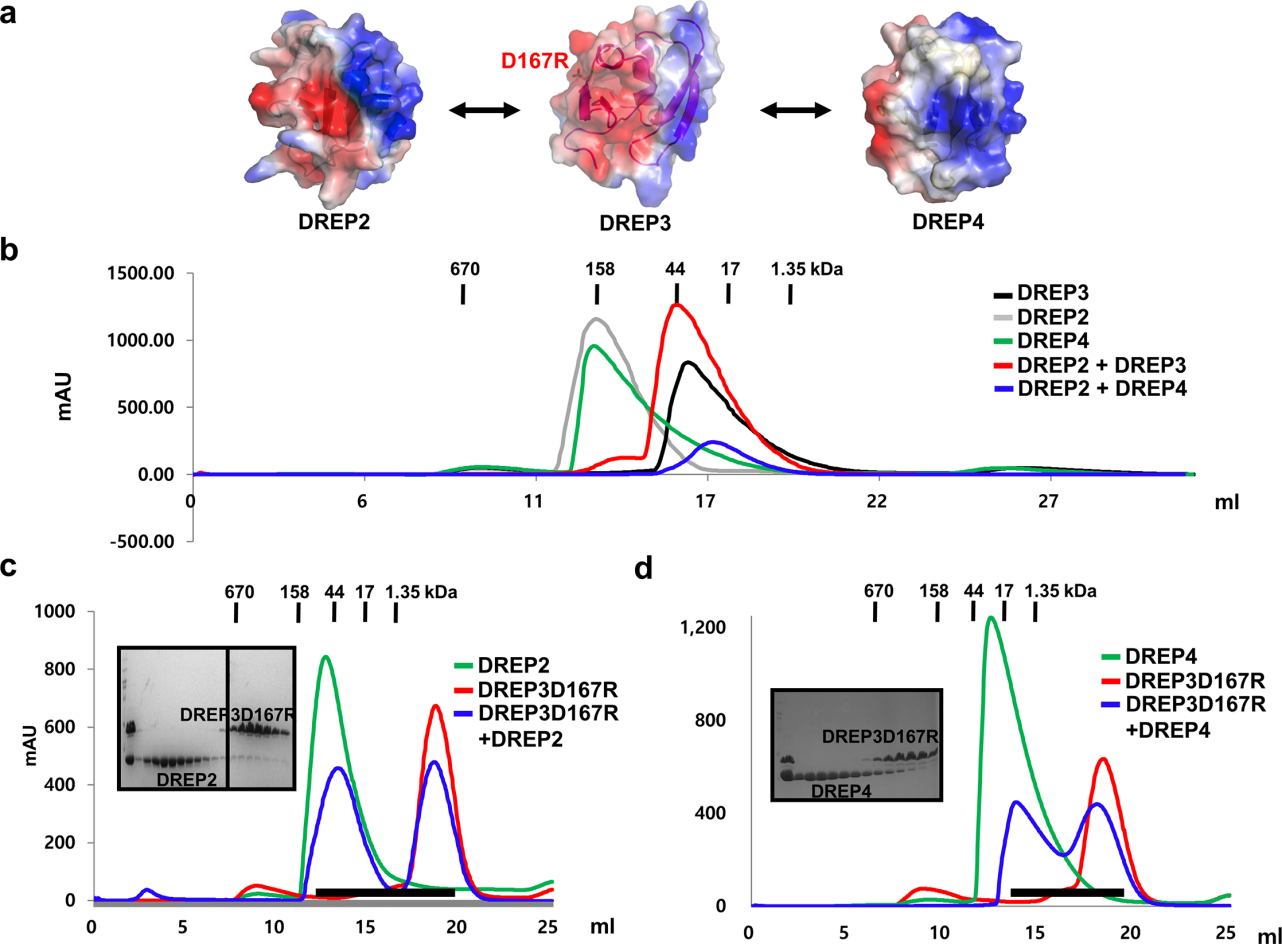
Acidic surface of DNA fragmentation factor 45 (DFF45)-related protein 3 (DREP3) cell death-inducing DFF45-like effector (CIDE) is involved in interaction with DREP2 CIDE and DREP4 CIDE (a) Known interaction partners of DREP1. (b) Size-exclusion chromatograms of CIDE domains of DREP2, DREP3, and DREP4, and mixtures of DREP2 CIDE with DREP3 CIDE and DREP4 CIDE. C. Size-exclusion chromatograms of DREP2 CIDE, DREP3D167R, and the mixture of both. SDS-PAGE-loaded fractions (black bar) of mixture of DREP3D167R and DREP2 CIDE from size-exclusion chromatography are shown on left side. (d) Size-exclusion chromatograms of DREP4 CIDE, DREP2D167R, and mixture of DREP3D167R and DREP4 CIDE. SDS-PAGE loaded fractions (black bar) of mixture of DREP3D167R and DREP4 CIDE from size-exclusion chromatography are shown on left side.

Both DREP2 CIDE and DREP4 CIDE formed a highly oligomeric complex in solution that was eluted in the size-exclusion chromatography around 12–14 mL, in contrast to DREP3 CIDE that formed a homodimer, which eluted around 16–17 mL (Fig 5A and 5B). It has been shown that the oligomeric DREP2 CIDE and DREP4 CIDE peak disappears following the formation of a complex with DREP3 CIDE and subsequent formation of a heterodimer [20]. The complex peaks were observed around 17 mL in the size-exclusion chromatography (Fig 5B) [20]. To analyze the interaction with the mutant DREP3D167R, which was disrupted on the acidic surface side of DREP3 CIDE, we performed size-exclusion chromatography. The profile showed that DREP3D167R was not able to form a complex with DREP 2 CIDE, as indicated by a homo-oligomeric DREP2 peak around 12–14 mL (Fig 5C). The SDS-PAGE showed two proteins that migrated separately, supporting the data from the size-exclusion chromatography (Fig 5C). After analyzing the interaction between DREP3D167R and DREP2 CIDE, we investigated whether the mutant interacted with DREP4 CIDE using the same mode and binding side. To accomplish this, we performed size-exclusion chromatography again, followed by SDS-PAGE and checked the position of the peak and co-migration with purified DREP4 CIDE and DREP3D167R. The reduced complex peak in the size-exclusion chromatography profile and the dissociated protein bands in the SDS-PAGE indicate that DREP3D167R did not interact with DREP4 CIDE (Fig 5D). These data indicate that the acidic surface of DREP3 interacted with both DREP2 and DREP4.

**Fig 6:**
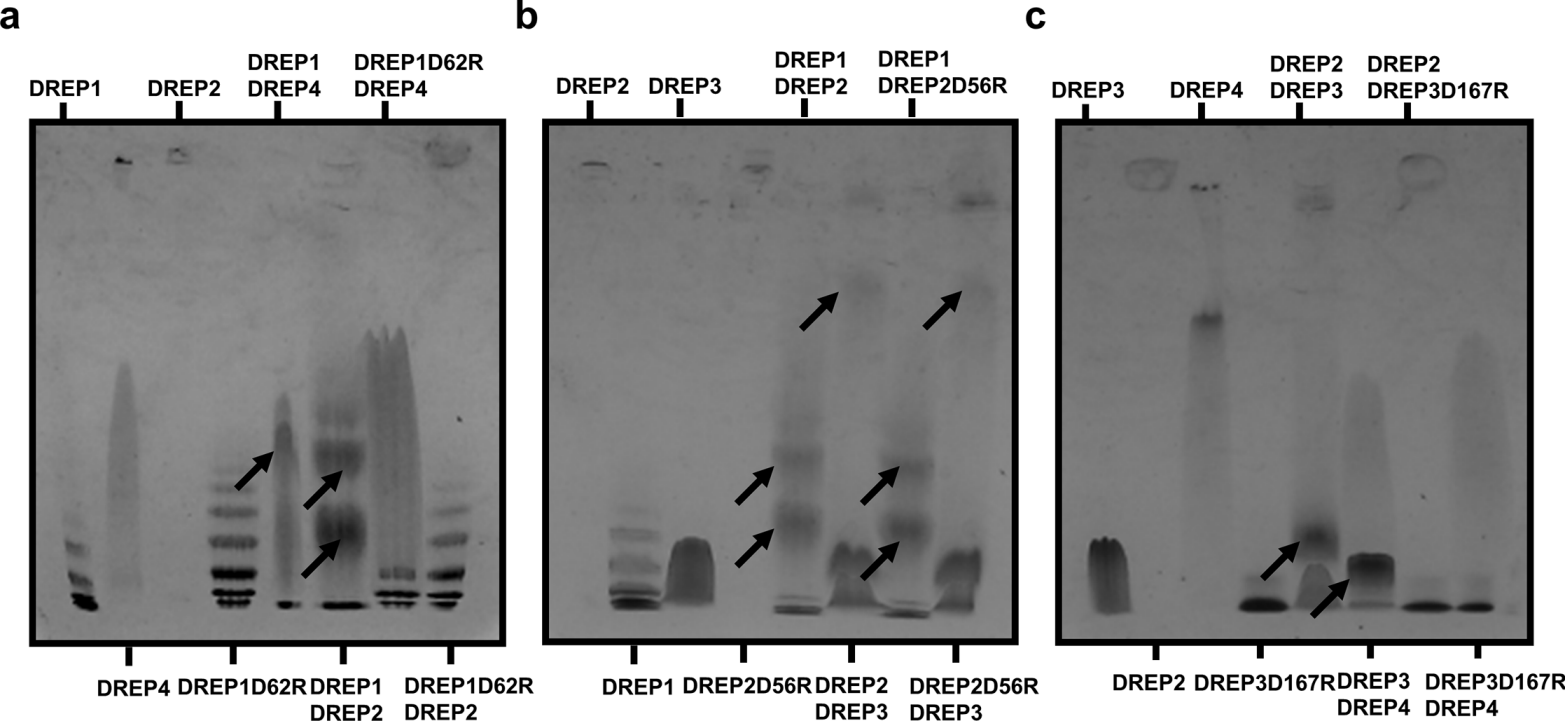
Native polyacrylamide gel electrophoresis (PAGE) to determine confirmation of mutation effects. Mutation effects on (a) DREP1 CIDE, (b) DREP2 CIDE, and (c) DREP3 CIDE. Loaded proteins on native PAGE gel are indicated. Newly formed complex bands are indicated by black arrows.

The specific bipartite interactions between the DREP proteins were also confirmed by a native PAGE analysis. Although the mixtures of DREP1 CIDE and DREP4 CIDE, and DREP1 CIDE and DREP2 CIDE produced complex bands on the native PAGE, the mixture of DREP4 and DREP1D62R, and DREP2 and DREP1D62R did not (Fig 6A). In contrast, the mixtures of DREP1 CIDE and DREP2D56R, and DREP3 CIDE and DREP2D56R, still produced complex bands on the native PAGE (Fig 6B). This result strongly supports the notion that the acidic surface side of DREP1 CIDE formed by D62 is crucial to its interaction with the basic surfaces of both DREP4 and DREP2. However, the acidic surface of DREP2 CIDE was not involved in the interaction with DREP1 CIDE. In addition, although the mixtures of DREP2 CIDE and DREP3 CIDE, and DREP3 CIDE and DREP4 CIDE produced complex bands, the mixture of DREP2 and DREP3D167R, and DREP4 and DREP3D167R did not (Fig 6C). This result supports the notion that D167 on the acidic surface of DREP3 is crucial to its interaction with both DREP2 and DREP4.

**Fig 7:**
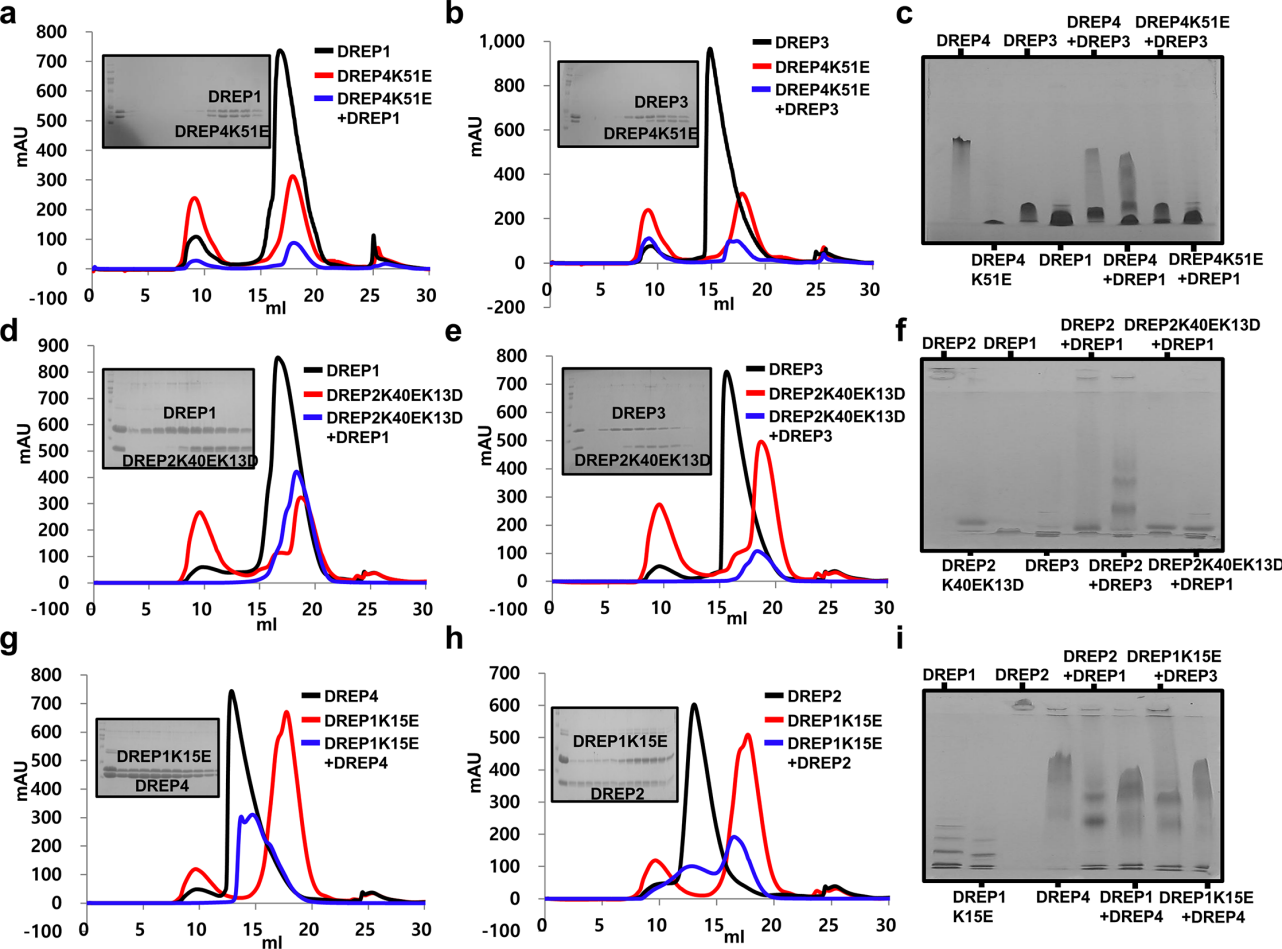
Basic surface of CIDE domains of DREP4 and DREP2 is involved in interaction with their interaction partners, while basic surface of DREP1 CIDE is not involved in interaction with DREP4 and DREP2. (a) Size-exclusion chromatograms of CIDE domains of DREP1, DREP451E, and mixtures of DREP1 andDREP4K51E. SDS-PAGE-loaded fractions of mixture of DREP1 and DREP4K51E from size-exclusion chromatography are shown on left side of the peak (b) Size-exclusion chromatograms of DREP3, DREP4K51E, and the mixture of both. SDS-PAGE-loaded fractions of mixture of DREP4K51E and DREP1 from size-exclusion chromatography are shown on left side. (c) Native PAGE. Loaded proteins on native PAGE gel are indicated. (d) Size-exclusion chromatograms of DREP1, DREP2K40EK13D, and mixture ofboth. SDS-PAGE loaded fractions of mixture are shown on left side. (e) Size-exclusion chromatograms of DREP3, DREP2K40EK13D, and the mixture of both. SDS-PAGE loaded peak fractions of mixture are shown on left side. (f) Native PAGE. Loaded proteins on native PAGE gel are indicated. (g) Size-exclusion chromatograms of DREP4, DREP1K15E, and the mixture of both. SDS-PAGE loaded peak fractions of mixture are shown on left side (h) Size-exclusion chromatograms of DREP2, DREP1K15E, and the mixture of both. SDS-PAGE loaded peak fractions of mixture are shown on left side (i) Native PAGE. Loaded proteins on native PAGE gel are indicated.

To confirm the experimental results performed with the acidic patch mutants, we also generated basic side-disrupted mutants of DREP1 (DREP1K15E), DREP2 (DREP2K40EK13D), and DREP4 (DREP4K51E) based on the sequence alignment and performed the same experiments, size-exclusion chromatography and native-PAGE, that have been applied for interaction analysis with acidic side-disrupted mutants. The experimental results showed that DREP4K51E did not interact to both DREP1 and DREP3 (Fig 7A, 7B and 7C). Same as DREP4K51E, basic patch-disrupted mutant of DREP2, DREP2K40EK13D, also failed to form a complex with both DREP1 and DREP3 (Fig 7D, 7E and 7F). These results are exactly matched with our expectation and confirmed our previous experimental results that have been performed with acidic patch-disrupted mutants of DREP4 and DREP2. In the case of basic side-disrupted mutant of DREP1, DREP1K15E, it still formed stable complex with both DREP4 and DREP2 on the size-exclusion chromatography followed by SDS-PAGE (Fig 7G and 7H). Native-PAGE, which showed newly produced bands at the samples prepared by mixing DREP1K15E with either DREP2 or DREP4, indicated that basic patch of DREP1 is not involved in the interaction with DREP2 and DREP4 (Fig 7I). Since acidic side surface of DREP1 was critical for the interaction with both DREP4 and DREP2, this result also supports our previous experimental result.

### Putative mode of interaction between DREP family CIDE domains

**Fig 8:**
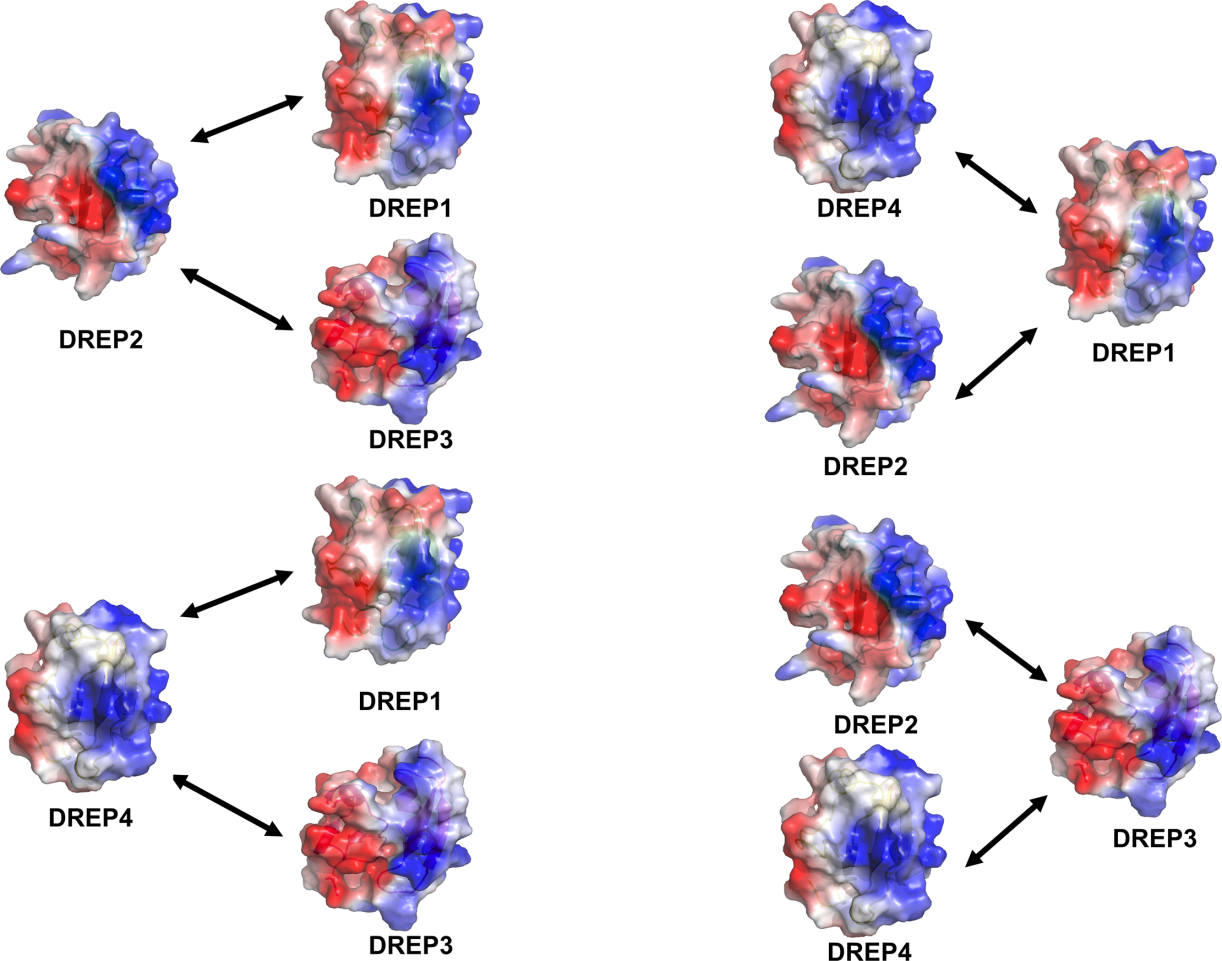
Predicted mode of interaction between DREP family proteins. Arrows indicate interactions. Bipartite surfaces are indicated in red and blue for acidic and basic surfaces, respectively.

Recent intensive biochemical studies revealed that CIDE domain-containing proteins in the fly, DREP1–4, interact with each other through specific binding partners via CIDE-CIDE interactions [20]. DREP1 interacts with DREP2 and DREP4, DREP2 interacts with DREP1 and DREP3, DREP3 interacts with DREP2 and DREP4, and DREP4 interacts with DREP3 and DREP1. Although the cyclic interaction between DREP family proteins via the CIDE domain, which has conserved charged residues on its opposite surfaces has been demonstrated, the interaction modes are not known. In this study, we identified the binding modes of interaction of DREP family proteins, using biochemical and mutational studies. DREP1 CIDE uses its acidic charged surface to interact with both DREP2 CIDE and DREP4 CIDE (Fig 8). Similarly, the acidic surface of DREP3 CIDE interacts with the basic surface of both DREP2 and DREP4 (Fig 8). This indicates that the basic surface of DREP2 CIDE interacts with the acidic surface of both DREP1 CIDE and DREP3 CIDE, and the basic surface of DREP4 CIDE interacts with the acidic sides of both DREP1 CIDE and DREP3 CIDE (Fig 8). We also found that the highly homo-oligomeric forms of DREP2 CIDE and DREP4 CIDE were disrupted by interaction with DREP1 CIDE or DREP3 CIDE. The binding affinities of the heterodimeric interactions were possibly higher than those of the homo-oligomeric interactions of DREP2 CIDE and DREP4 CIDE were. Finally, the functional relevance of this mode of interactions between DREP family proteins in the fly would have to be further elucidated in future.
